# A Standardized Peer Review Program Improves Assessment and Documentation of Child Sexual Abuse

**DOI:** 10.1097/pq9.0000000000000522

**Published:** 2022-01-21

**Authors:** Suzanne P. Starling, Kimberly A. Martinez, Lori D. Frasier

**Affiliations:** From the * Children’s Hospital of The King’s Daughters, and Eastern Virginia Medical School, Norfolk, Va.; † Midwest Regional Children’s Advocacy Center, Edina, Minnesota, and Children’s Hospitals and Clinics of Minnesota, Minneapolis, Minn.; ‡ Penn State Hershey Medical Center, Hershey, Pa.

## Abstract

**Introduction:**

The objective was to assess the impact of interventions associated with ongoing expert peer review on the quality of child abuse medical providers’ written and photograph documentation in child sexual abuse cases.

**Methods:**

Pediatricians participated in a HIPAA compliant blind peer review process on a web platform developed to provide the American Board of Pediatrics maintenance of certification. Participants submit sequential photograph and written documentation of child sexual abuse examinations over 1 year. Documentation includes genital examination descriptions and interpretation of findings. Reviewers evaluate the photographic quality and written documentation of examination findings utilizing a numerical rating system. Each case cycle is reviewed by one of four experts in child abuse who received training in a uniform evaluation process. Reviewers follow each case throughout three cycles of documentation, selecting from several interventions that have been customized to address the quality issues noted. The third and final cycle includes summary comments from the reviewer.

**Results:**

Forty-one participants completed the program at the time these data were collected. A paired *t* test analysis of the combined scores of the three measures, such as Image Quality, Quality of Written Documentation, and Accuracy of Exam Interpretation, showed a statistically significant improvement (*P* < 0.001) between the first and last sets. In addition, each of the individual measures was statistically significant between the first and last case sets with a *P* value of <0.05 for each.

**Conclusion:**

Peer review with interventions customized to address quality issues improved the quality of the assessment and documentation of child sexual abuse evaluations.

## INTRODUCTION

### Problem Statement

Over the past 25 years, the field of Child Abuse Pediatrics has developed a robust body of literature describing normal and abnormal findings in genital examinations.^1–6^ Despite this literature, clinicians evaluating child sexual abuse may make errors in diagnosis. Several studies have demonstrated that case history can influence inexperienced examiners, leading to an overinterpretation of the examination results indicating trauma.^7^ Examiners may also misinterpret physical findings, leading to overdiagnosing child sexual abuse based solely on inaccurate medical findings.^7–14^

### Available Knowledge

Overcalling physical findings diagnostic of sexual abuse can sway case investigators in how they proceed with their case investigation and potentially influence the judicial outcome through misrepresentation of examination findings. Unfortunately, false beliefs and significant misunderstandings that sexual abuse cases will have physical evidence exist. For example, in the nonacute prepubertal populations, less than 5% of girls alleging genital penetration have injuries resulting from sexual abuse,^1,3,15^ and fewer than 1% of children alleging anal perpetration have positive anal findings from abuse.^3,6,16^ Therefore, the judicial case should not rest solely on medical evidence. Instead, the foundation of a thorough child abuse evaluation consists of the accurate description of the physical examination accompanied by quality photographic documentation coupled with the child’s statements and other corroborating evidence.

### Rationale

For child sexual abuse cases, a comprehensive medical evaluation consists of thorough written documentation, high-quality interpretable photograph documentation of anogenital findings, and accurate interpretation of the examination,^6,12,17^ resulting in improved quality of legal evidence.

Oversight of child sexual abuse cases, through the peer review process, is an essential part of the accuracy of diagnosis,^11–16,18–23^ reduces bias in the examination and reduces the influence of history in cases where examination findings are normal.^16,20^ Ensuring the most accurate diagnosis improves the health and safety of this population of children. Peer review serves as a mode of continuing education to ensure quality medical evaluations. Effective peer review cannot occur without written and photo documentation that adequately represents the medical evaluation. Expert review is valuable in judicial proceedings to demonstrate nonbias in clinical judgment.

### Specific Aims

The Midwest Regional Children’s Advocacy Center (MRCAC) is a federally funded Department of Juvenile Justice, Delinquency and Prevention program that provides training and technical assistance to medical providers who evaluate child abuse. In addition, the MRCAC provides the project platform and administration of *myQIportal*, an American Board of Pediatrics approved Part IV Maintenance of Certification (MOC) activity. This project aims to assess and improve the medical diagnosis of child sexual abuse and increase consistency of evaluation through improvement in the quality of photographic documentation, written documentation, and diagnostic accuracy.

## METHODS

### Context

A quality improvement (QI) project was undertaken to assess and improve the quality of written and photographic image documentation and overall diagnostic accuracy of physicians who provide suspected child sexual abuse medical evaluations. Recruitment to the project included advertising in medical and partner newsletters and national child abuse conferences, direct emails, and targeted listservs and websites. Part 4 MOC credit is available for the physician participants. Based upon the principles of the IHI Breakthrough Series collaborative model,^23^ the authors built a project offering interwoven learning opportunities accompanied by interventions, helping clinicians build upon their existing skills to improve clinical practice.

No scoring system existed at the time this project was developed to assess written and photo documentation of child abuse evaluations; therefore, the authors developed a scoring system to assess each aspect of child sexual abuse evaluations based on their experiences as child abuse educators, practitioners, and years of participation in formal and informal peer review.

Participants submit three case sets, consisting of five consecutive child sexual abuse evaluations, for review and are scored on three components: Image Quality, Written Documentation Quality, and Examination Interpretation Accuracy (Table 1). Interventions are then assigned. Participants are required to submit at least two anogenital photographs per case, with the intent for these images to capture the essential elements of the anogenital examination. Typical child sexual abuse examination consists of at least two images to adequately document both hymenal and anal findings.^16,17,24–28^ If abnormal findings are noted, additional images would be warranted to document the findings so that the examination can be peer-reviewed without requiring the child to undergo a second examination. Alternatively, some sites utilize video colposcopy to demonstrate the entire exam rather than digital still photographs. Cases that do not have at least two photographs may receive a score of “0” due to the inability to review the anogenital examination adequately. The scoring tool utilized did not include (1) assessment of forensic evidence collection in acute sexual abuse cases; (2) follow-up care; or (3) explicit requirements that all essential anatomical structures be visible to ensure a complete examination (this is addressed within the scoring system). In total, participants submitted three case sets for review and intervention (total of 15 evaluations) throughout the project.

**Table 1.** Standardized Scoring System with Interventions

**Measure:** Quality of Written Documentation		
(possible score 4 points)		
Definition of measure:		
Quality of the written documentation of the exam findings in child abuse cases		
Improvement target value:		
Improve the score on this measure after intervention		
Calculation:		
	Points	
Identifies all pertinent findings in photograph	0	1
Accurately describes all findings	0	1
Accurately describes location of findings	0	1
Does not describe normal finding as abnormal	0	1
Acceptable: 4 points (high score)		
Unacceptable: 0–3 points (low score)		
Intervention:		
PowerPoint presentation on improving documentation		

**Measure:** Image Quality		
(possible score 5 points)		
Definition of measure:		
Quality of the submitted photographic images in the support of the child abuse diagnosis		
Improvement target value:		
Improve the score on this measure after intervention		
Calculation:		
	Points	
Color-represents natural, expected skin tones	0	1
Brightness/contrast, delineation of shadows	0	1
Focus-sharpness, delineation of the findings	0	1
Composition, subject in field	0	1
Adequate representation of described finding	0	1
Excellent: 4–5 points (high score)		
Acceptable: 2–3 points		
Unacceptable: 0–1 points (low score)		
Interventions:		
Viewing textbook chapter on photography		
Two videos on photography: basic photography and photo documentation of the female genitalia		

**Measure:** Accuracy of Examination Interpretation		
(possible score 1 point)		
Definition of measure:		
Accuracy of the diagnostic interpretation child abuse cases		
Improvement target value:		
Improving the score on this measure after intervention		
Calculation:		
		Points
Reviewer agrees with the interpretation of the findings		1
Reviewer disagrees with the interpretation of the findings		0
Reviewer cannot review the findings		0
Acceptable: 1 point (high score)		
Unacceptable: 0 points (low score)		
Interventions:		
Introduction to child sexual abuse medical evaluation video		
Advanced evaluation of child sexual abuse video		
One or more peer-reviewed articles on diagnosis:		
• Medical evaluation of suspected child sexual abuse: 2011 update		
• Updated guidelines for medical assessment for the medical assessment and care of children who may have been sexually abused: 2016		
• Guidelines for medical care of children evaluated for suspected sexual abuse: an update for 2018		

### Measures

**Fig. 1.**
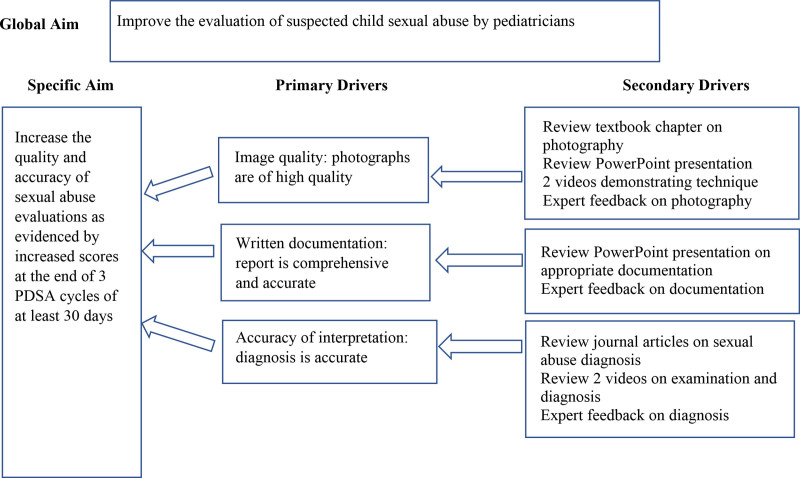
Key driver diagram.

Key drivers for written and photograph documentation and overall assessment interpretation were identified based on the years of experience of the CAP authors of this study and previous QI projects (Fig. 1).

Numeric scores are awarded for each category, and interventions are assigned based on the score received. For instance, a low score in photograph documentation would warrant an intervention for photography, whereas a high score in written documentation would not warrant intervention in that cycle. After reviewing each case set, participants receive the numeric scores, detailed written reviews of the photograph and written documentation with specific suggestions for improvement, and assigned interventions via the electronic platform. All participants receive interventions between the first and second case sets.

In some cases, initial scores are very high, and interventions are assigned based on the lowest score achieved, even if that score falls within the parameters of a good score. In such cases, the participant is expected to possess high-level skills in the diagnostic process and would rate consistently high scores throughout the process. A very high score (9 or 10) demonstrates little room for improvement with that specific case. A low score is a score below 6 (see Table 1).

After completing the assigned interventions, participants are permitted to upload their second case set for review and scoring. During the evaluation of the second set of cases, the reviewers can access the participants’ previous scores and ascertain if they are improving in the desired skill set. Then, further interventions are assigned based on the reviewer’s scoring assessment of the skills (see Table 1). After completing the second intervention(s), participants submit a final case set for review, receiving a final score and summary statement.

Three board-certified child abuse pediatricians (CAPs), each with more than 15 years experience in the field, were selected as initial reviewers for the project; one additional CAP reviewer was added to help manage the increased participant caseload. All reviewers developed the national standards for medical diagnosis of child abuse and routinely provided education to physicians regarding child sexual abuse. The project leader trained all four reviewers in standardized reviews to ensure consistency in scoring and evaluation. This training also served to establish interrater reliability. Participants are assigned to a single reviewer to receive consistent evaluation throughout the process and minimize interrater reliability issues. Reviewers only assess their assigned case sets, allowing them to follow the progress of the individual clinician. The reviewer and clinician are blinded to one another to eliminate any potential bias in the scoring of the case submissions. By knowing the previously assigned interventions, reviewers can layer educational interventions for the submitting clinicians.

Data from all participants who submitted three complete case sets and implemented the interventions at the time of this study were analyzed. In addition, participants were benchmarked against themselves to determine if their scores improved after the interventions. The use of paired t-tests and any differences was considered significant if the test statistic probability was at or below 0.05.

The *myQIportal* program was initially hosted on a platform developed by Visual Share, later acquired by XIFIN, Inc. (San Diego). The XIFIN ProNet framework, applications, and data center comply with the Health Insurance Portability and Accountability Act (HIPAA) to ensure all patient information remains protected and confidential. All XIFIN electronic features and functionality adhere to strict privacy and security rules regarding Protected Health Information at three levels: administrative controls, physical safeguards, and technical safeguards for authentication and encryption.

### Ethical Considerations

The Eastern Virginia Medical School, Institutional Review Board, reviewed the project and considered it a QI initiative and not research involving human subjects.

Standard of care in the field includes examining photos in all cases of suspected child sexual abuse. Before the patient evaluation, all patients must permit to have images taken. Before submission to this platform, all images are required to be de-identified with no HIPAA information included. Both images and digital cameras and/or video colposcopes utilized to capture the images are safeguarded under HIPAA guidelines.

### Interventions

For Image Quality, photographic quality is scored by components of color, brightness/contrast, focus, composition, and adequate representation of the pertinent findings (Table 1). Five points are possible. Scores of 0–2 are considered unacceptable and warrant interventions to improve photographic skills and image quality, whereas cases with scores of 3–4 could also warrant photographic interventions. Reviewers also provide participants with a detailed narrative description of any issues with their images, designed to help improve the quality of the images. Interventions for Image Quality include reading a textbook chapter on photography^17^ or viewing one of two videos on photography explicitly produced for this project: Photo documentation of the Female Genitalia,^29^ and Basic Photography by John Melville, MD.^30^

For Quality of Written Documentation, participants are scored on identifying pertinent anatomy in the photographs, accurately describing all findings with correct locations, and avoiding misdiagnosis of a finding. Four points are possible; a score less than 3 is considered unacceptable. In addition, participants receive a detailed narrative description of any issues with their written documentation. The intervention specific to Documentation of Child Sexual Abuse is a PowerPoint presentation on improving documentation written by the authors.^31^

Examination Interpretation Accuracy was developed using a system benchmarked to nationally published standards of diagnostic criteria.^1,6,32,33^ Participants receive a score of 0 or 1 reflecting reviewer agreement or disagreement with the diagnosis based on the national standards. In some cases, the images are inadequate for review, and the participants receive a zero score for that case. Interventions for Accuracy of Examination Interpretation are designed to improve diagnostic skills and included reviewing three peer-reviewed research papers or one of two video presentations on sexual abuse diagnosis produced by the MRCAC for the education of participating medical providers on child sexual abuse: Introduction to Child Sexual Abuse Medical Evaluation by Lori Frasier, MD^35^ or Advanced Evaluation of Child Sexual Abuse by Suzanne Starling MD.^36^

In addition to interventions based on numeric scores, all participating medical providers receive general information on the basic tenets of QI to review at the beginning of their submission process. Participants are directed to the IHI QI tutorial^37^ and a journal article on QI.^38^

## RESULTS

Forty-one physicians participated in the program during the first 6 years of the program. Thirty-three were board-certified CAPs, and eight were general pediatricians that conduct child abuse medical evaluations. One of the participants had practiced less than 1 year, eight of the physicians had practiced 1–5 years (3 general pediatricians, 5 CAPs), nine practiced 6–10 years (eight CAPs and one general pediatrician), eight CAPs practiced 11–15 years, and the remaining 15 physicians practiced more than 15 years (3 general pediatricians and 12 CAPs). Eight physicians saw less than 50 children/year (1 general pediatrician and 7 CAPs), nine saw between 51 and 100 children/year (1 general pediatrician and 8 CAPs), 11 saw between 101 and 250 children/year (3 general pediatricians and 8 CAPs), and another 11 (2 general pediatricians and 9 CAPs) saw more than 250 children/year for suspected child sexual abuse. Two physicians did not indicate how many children they evaluated annually for suspected child sexual abuse (one MD who works in an emergency room and one CAP). Six sites are considered rural, whereas the remaining sites are all located in urban areas.

**Fig. 2.**
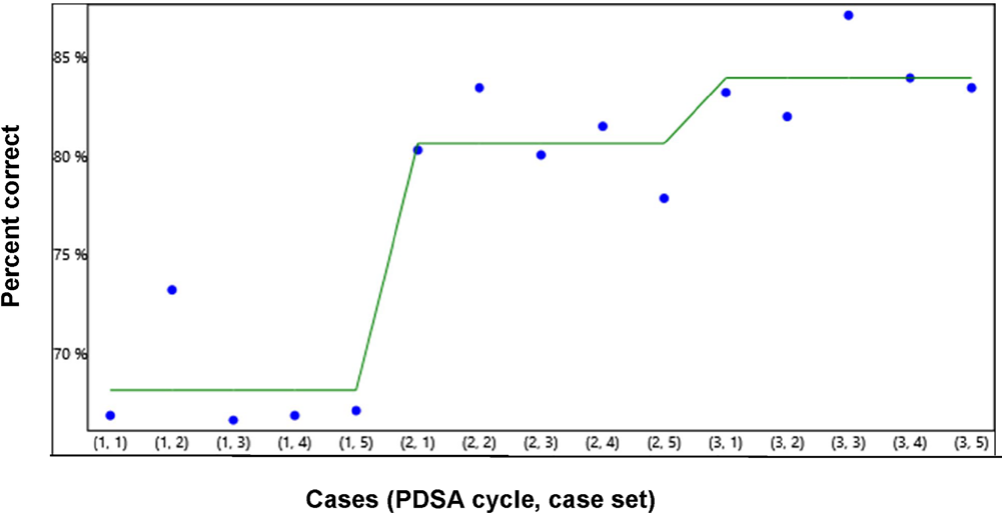
Run chart demonstrating the overall improvement mean scores over three case sets.

Figure 2 is a run chart demonstrating the mean scores of all 41 participants for the 15 case submissions. Thirty-three of 41 (80.5%) physicians showed overall improvement when all categories were considered. Overall, we found significant changes in all three categories between the first and last set submissions (*P* < 0.001). Participants showed the most significant change in their cumulative scores of all three measures between the first and second sets and between the first and last sets. There was a decrease in the percentage of change between the second and third sets (Fig. 2).

**Fig. 3.**
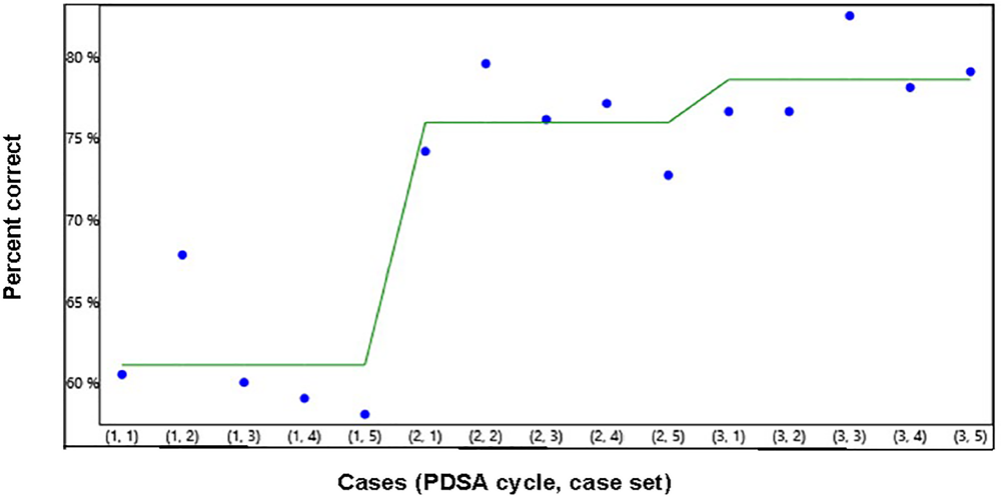
Run chart demonstrating the improvement in image quality mean scores over three case sets.

Image Quality scores showed the most variability (Fig. 3). Twenty-eight of 41 participants (68%) showed improvement, with a *P* value of <0.05. Weighted image scores were the lowest of the three scoring categories, and all participants received interventions for photography during their participation in the project. For image quality, we found improvement in quality between case set submissions one and three, consisting of magnified and focused images with a good demonstration of the entire exam, including any abnormal findings. Many participants’ images were unfocused or incomplete images that did not demonstrate the entire genital examination, prohibiting an assessment of the examination. This photographic error improved over case sets.

**Fig. 4.**
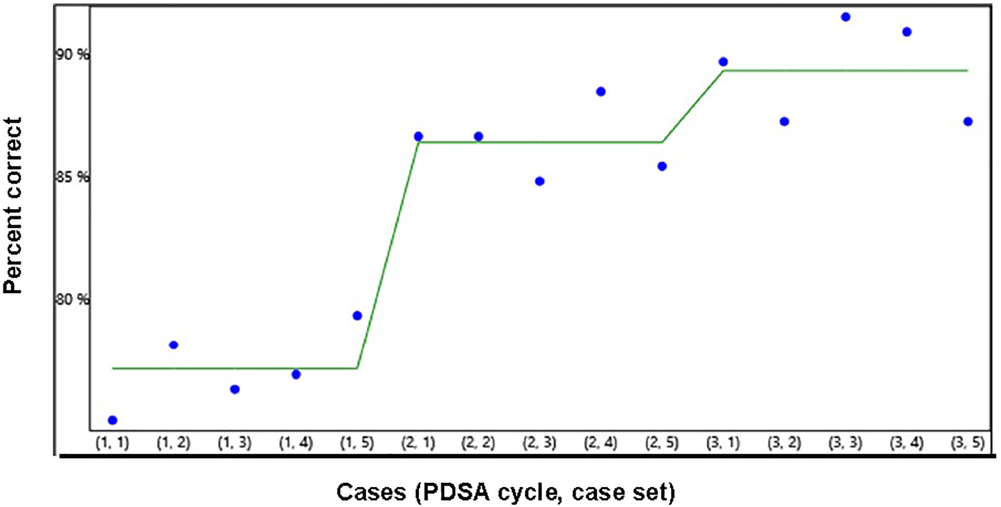
Run chart demonstrating the improvement in written documentation mean scores over three case sets.

Quality of Written Documentation scores showed much less variability for the cases submitted. For written documentation, 22/41 showed improvement (53.6%), with a *P* value of <0.05 (Fig. 4).

**Fig. 5.**
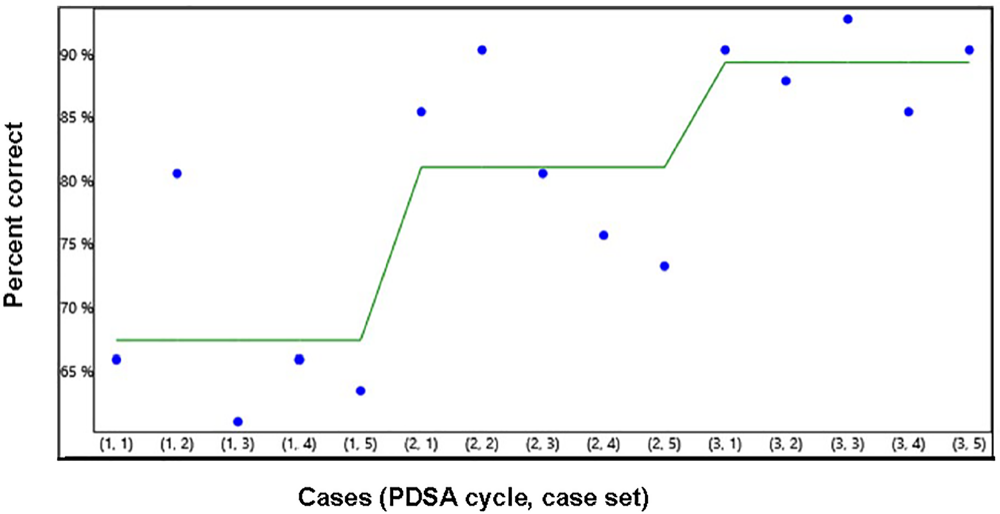
Run chart demonstrating the improvement in diagnostic accuracy, mean scores over three case sets.

Accuracy of Examination Interpretation was comparable to written documentation quality. Twenty-three of 41 (56.1%) showed improvement with a *P* value of <0.05 (Fig. 5).

Of the 41 complete submissions reviewed, two participants scored 100% on their initial submissions. As expected, these two participants did not show improvement since they had already attained the maximum score. However, they maintained high scores throughout the process, decreasing by only 2 points overall.

## DISCUSSION

These data show that the participants in the peer review process significantly improved their assessment and documentation in child sexual abuse cases. Overall, significant changes in all three categories were measured between the first and last case sets, suggesting that the intervention is cumulative. Some combination of both the first and second set of interventions was necessary to cause improvement.

### Interpretation

Diagnostic accuracy in child abuse pediatrics has health and legal ramifications and must be as accurate as possible. Many of the initial images submitted to the system were of poor quality. Poor photograph documentation affects the ability to peer review cases and ultimately leads to many errors in diagnosis. After the intervention, the photographs improved, demonstrating that targeted intervention can improve this skill. This MOC project is ongoing, and cases are continually enrolled, so we expect participants to continue to show QI through this initiative. The results of this project also have opened a national dialogue around the subject of photograph documentation, resulting in improved communication among colleagues regarding methods to obtain the best images possible for each case. Further efforts such as these will need to be made nationally and internationally to continue to improve photographic quality in our field, including having all cases of suspected abnormal findings subjected to expert peer review. The National Children’s Alliance (NCA) is moving in this direction, updating their medical standard to change from having at least 50% of all abnormal cases expert peer-reviewed to requiring 100% of abnormal examinations be expert peer-reviewed.^39^

There was a poor understanding of the research used to diagnose sexual abuse in some cases. This project strives to teach providers only to diagnose what can be supported by the literature. Future research can focus on these areas of diagnosis that remain inadequately explored.

Several lessons were learned throughout this project. Modifications were made to the interventions assigned for image quality when the trends were not showing improvement in images after the second intervention. Different types of intervention may appeal to different individuals, and different media are utilized to reach all learners. Further efforts such as these will need to be made to improve photographic quality in all aspects of our field, not just in the arena of child sexual abuse. To address this, a physical abuse QI initiative (Improving Documentation in Child Physical Abuse Medical Evaluations) addressing these issues was designed and is in use through a collaborative effort between the MRCAC and the American Academy of Pediatrics, available for 25 Part 4 MOC credits (Grant #2019-CI-FX-K004 awarded by the Office of Juvenile Justice and Delinquency Prevention, US Department of Justice). The results of this project were outlined in a 2021 publication.^40^

Individuals who started with high scores did not remain static over time and often decreased in scores. These individuals started with such high scores that perfection would have been the only way to maintain their level. This observation suggests that their decrease in scores reflects their awareness that they would “pass” the project based on the strength of their initial scores resulting in a subsequent reduction in the fine attention to detail rather than an actual decrease in the quality of their work. A longer cycle involving more than three sets of images may have resulted in different outcomes over time.

### Limitations

This initiative has several limitations:

1. The program is only accessible to pediatricians seeking MOC QI points. Therefore, the project design is explicitly for pediatricians who perform genital photography as part of the assessment for child sexual abuse. This design results in a very experienced sample of participants, most of whom were maintaining certification for their child abuse subboard certification.
2. Participants in this MOC activity may have been mainly motivated to complete the project for the MOC points without intending to make substantive improvements in care. Whether each provider completed the assigned interventions is not possible within this project’s scope, and not every provider received interventions on every review. For example, all providers received interventions on photography, but not all received interventions on written documentation and assessment.
3. This project has no control over whether participants are involved in other peer reviews or educational opportunities to improve their performance.
4. At the time this project was undertaken, no standardized scoring tool existed. However, two known projects have been launched since then, both assessing child physical abuse imaging with scoring tools. The MRCAC, launched in 2016, hosts one. The other project is from the Child Advocacy and Protection Services in Milwaukee, Wis., which launched a study in May 2018 to assess a tool they have designed as part of a QI initiative for Part 4 MOC credit.^41^ Their study is very similar to the photo documentation component of this project; the results are still pending.
5. Interrater reliability was not conducted, which would have ensured that reviewers continued to interpret the defined categories consistently. Intermittent interrater reliability testing would be beneficial for this project and will be implemented in the future.
6. There may be potential implicit bias in the further case set scoring due to reviewers having access to the prior case set scores and assigned interventions. Still, this potential bias is offset by the reviewers’ ability to layer educational resources designed to benefit the submitting physician.

## CONCLUDING SUMMARY

This study demonstrated that peer review with custom-selected interventions is effective. In addition, improvement in the quality of the photodocumentation of child sexual abuse assessments and improved written quality and accuracy of examination interpretation were noted.

Improved skills from the interventions are expected to be sustained, replicated, and disseminated among medical colleagues, including those not active participants in this project. The participants have continued access to the educational material provided and can draw on this information as they progress through subsequent cases. Once the appropriate documentation is modeled and refined for one participant, examiners can share this information within their practice groups and influence how an entire group of physicians and their medical colleagues diagnose and document abuse. Through peer review participation, clinicians can share their knowledge and influence their colleagues concerning improvement in written and photodocumentation and improvement in diagnostic impression.

Although such a secure system of anonymous peer review on a national level may be challenging to duplicate locally, the basic concept of case review and image improvement is generalizable to the entire field. It is possible to demonstrate the principles of targeting improvement of photography and documentation on a local level. We encourage local and regional peer review as a means of QI in the field.

By improving diagnostic practices, medical providers can improve the community response to child sexual abuse. Providing accurate assessments to the agencies involved in protecting children and prosecuting offenders will result in improved safety of children.

## ACKNOWLEDGMENTS

The authors thank Patricia Goede, PhD, Vice President of Clinical Informatics at XIFIN, who developed the platform utilized for this program. We also thank Michael Finch, PhD, Research Projects and Analytics Manager, who provided data analysis and statistical interpretation. John Melville, MD, assisted with the data presentation.

## DISCLOSURE

The authors have no financial interest to declare in relation to the content of this article.
